# Leptin stimulates tissue rat mast cell pro-inflammatory activity and migratory response

**DOI:** 10.1007/s00011-018-1171-6

**Published:** 2018-07-17

**Authors:** Paulina Żelechowska, Justyna Agier, Sylwia Różalska, Magdalena Wiktorska, Ewa Brzezińska-Błaszczyk

**Affiliations:** 1 Department of Experimental Immunology, Faculty of Health Sciences, Medical University of Lodz, Lodz, Poland; 2 Department of Industrial Microbiology and Biotechnology, Faculty of Biology and Environmental Protection, University of Lodz, Lodz, Poland; 3 Department of Molecular Cell Mechanisms, Faculty of Health Sciences, Medical University of Lodz, Lodz, Poland

**Keywords:** Mast cells, Adipocytokines, Leptin, Inflammation

## Abstract

**Objective:**

The aim of this study was to determine whether leptin, a member of the adipocytokines involved in immune and inflammatory response regulation, may influence some aspects of mast cell biology.

**Materials and methods:**

Experiments were done in vitro on fully mature tissue rat mast cells isolated from the peritoneal cavity, and leptin was used at concentrations 0.001–100 ng/ml. The effect of leptin on mast cell degranulation (histamine release assay), intracellular Ca^2+^ level (fluorimetry), pro-inflammatory mediator release (ELISA technique), surface receptor expression (flow cytometry and confocal microscopy), and migration (Boyden microchamber assay) was estimated.

**Results:**

Leptin was found to stimulate mast cells to degranulation and histamine release. It induced the intracellular Ca^2+^ increase, as well. In response to leptin stimulation, mast cells generated and released cysLTs and chemokine CCL3. Leptin-induced upregulation of CYSLTR1 and CYSLTR2 surface expression was observed. Moreover, this adipocytokine stimulated mast cells to migratory response, even in the absence of extracellular matrix (ECM) proteins.

**Conclusions:**

Our observations clearly documented that leptin promotes the pro-inflammatory activity of mast cells, and it thereby engages these cells in the inflammatory processes.

## Introduction

Mast cells are long-lived resident cells and are widespread throughout connective tissues. These cells are typically most abundant near surfaces exposed to the environment, i.e., beneath the subepithelial surface of the skin and in the respiratory system, as well as in the gastrointestinal and genitourinary tracts. They are also found in close association with nerves, blood vessels, and smooth muscle cells [1]. Mast cells have the potential to secrete a wide range of biologically active mediators, cytokines, and chemokines. Upon activation, these cells rapidly release stored, cytoplasmic granule-associated mediators, such as histamine, chymase, tryptase, metalloproteinases (MMPs), heparin, and some cytokines and chemokines. Mast cells may secrete newly synthesized lipid derivatives, including leukotrienes (LTs), prostaglandins (PGs), thromboxanes (TXs), and a lot of cytokines, chemokines, and growth factors. It should be stressed, however, that these cells can generate and secrete mediators selectively: they may release either a vast array of products or distinct subsets of mediators [2, 3].

It is evident that various mast cell-derived mediators strongly influence the biological activity of adjacent cells and tissues [2, 4]. Hence, mast cells not only participate in maintaining body homeostasis and various physiological processes [1, 4, 5] but also play a role in the mechanisms of many pathological processes and are mainly well known for their involvement in allergic reactions [6, 7]. Mast cells are involved in both innate and acquired immune responses [8] and take part in the host defense against pathogens [9]. Undoubtedly, mast cells are widely recognized as principal effector cells of inflammatory processes since their mediators exert strong pro-inflammatory effects, but some of them also have anti-inflammatory and immunoregulatory effects. Thus, these cells affect different stages of both acute and chronic inflammation, including its initiation, maintenance, and resolution [10, 11].

Leptin, a 16 kDa non-glycosylated polypeptide, is mainly produced by adipocytes [12], although it is also found in immune cells, such as T cells, basophils, and mast cells [13–15]. Leptin is primarily known for its role as a hypothalamic modulator of food intake, body weight, and energy expenditure [16]. Interestingly, its circulating levels closely correlate with body mass index (BMI) as well as adipose tissue mass [17]. More and more data indicate that this adipocytokine is involved in regulating immune and inflammatory responses [18, 19], and it exerts predominantly pro-inflammatory effects [20]. There is also information that leptin may influence the course of the adaptive immune response [21].

Considering that mast cells are the key players in the course of immunological and inflammatory processes, and that leptin influences both of them, it seems to be of great importance to determine the direct effect of leptin on mast cell activity. There is currently a lack of data regarding direct action of this adipocytokine on mast cells. The present study examines the impact of leptin on mast cell degranulation, and the generation of substantial pro-inflammatory mediators. It also examines whether leptin affects the expression of CYSLTR1 and CYSLTR2, i.e., receptors for cysteinyl (cys)LTs, which act as powerful pro-inflammatory mediators in mast cells. It also analyses the effect of leptin on the mast cell migratory response.

## Materials and methods

### Materials

Dulbecco’s Modified Eagle Medium (DMEM) was purchased from Biowest (Kansas City, MO, USA). Hank’s balanced salt solution (HBSS), sodium bicarbonate, fetal calf serum (FCS), gentamicin, and glutamine were obtained from GIBCO (Gaithersburg, MD, USA). NaCl, KCl, MgCl_2_, CaCl_2_, 2-hydroxyethylpiperazine-N’-ethanesulphonic acid (HEPES), NaOH, glucose, HCl, *o*-phthaldehyde (OPT), compound 48/80, calcium ionophore A12387, Percoll®, hematoxylin, toluidine blue, trypan blue, bovine serum albumin (BSA), laminin from human placenta, phosphate-buffered saline (PBS), Triton™ X-100, and ethylene glycol-bis(2-aminoethylether)-N,N,N′,N′-tetraacetic acid (EGTA) were purchased from Sigma-Aldrich (St. Louis, MO, USA). Fluo-4 Direct™ Calcium Assay Kit was purchased from Thermo Fisher Scientific (Waltham, MA, USA). Black, 96-well cell culture plates were obtained from SPL Life Sciences Co. (Pocheon, Korea). Human plasma fibronectin purified protein was obtained from Merck Millipore (Billerica, MA, USA). Recombinant rat leptin, recombinant rat tumor necrosis factor (TNF), and normal goat IgG antibodies were obtained from R&D Systems (Minneapolis, MN, USA). Mouse anti-rat IgE monoclonal IgG1 antibodies (anti-IgE) were purchased from AbD Serotec (Oxford, UK). BD CellFIX™ was obtained from BD Biosciences (Benelux, NV, Belgium). Goat polyclonal IgG N-term antibodies against CYSLTR1 and CYSLTR2 were purchased from Santa Cruz Biotechnology, Inc. (Dallas, TX, USA). Alexa Fluor® 488-conjugated rabbit anti-goat polyclonal antibodies were obtained from Jackson ImmunoResearch Laboratories, Inc. (West Grove, PA, USA). Rat CCL3 and cysLT specific immunoassay kits were purchased from Cloud-Clone Corp. (Katy, TX, USA) and Cayman Chemical (Ann Arbor, MI, USA), respectively. The 48-well Boyden microchamber as well as the 8-µm-pore-size polycarbonate filters were purchased from Neuro Probe (Gaithersburg, MD, USA).

### Experimental animals

Mast cells were collected from the peritoneal cavities of female Wistar rats weighing 200–250 g, aged 3–4 months, bred in the animal quarters of the University of Lodz. Standard storage conditions for animals were provided, i.e., room temperature, a 12/12 h light/darkness regimen under artificial lighting. The rats were kept in metal cages, with five animals in each. The animals were fed with LSM Murigran granulated fodder for rodents and water ad libitum. All animal experimental procedures were carried out in strict accordance with the recommendations in the act on the protection of animals used for scientific or educational purposes. Protocols were approved by the Local Ethics Committee for Experiments on Animals in Lodz (the approval number 55/ŁB42/2016).

### Isolation of mast cells

Peritoneal cell suspensions were obtained from the peritoneal cavities by lavage with 50 ml of 1% HBSS supplemented with 0.015% sodium bicarbonate. After abdominal massage, the cell suspension was removed from the peritoneal cavity, centrifuged (1200 rpm, 5 min, 20 °C), and washed twice in complete (c)DMEM containing DMEM supplemented with 10% FCS, 2 mM glutamine, and 10 µg/ml gentamicin. To obtain purified mast cells, the cell suspensions were resuspended in 72.5% isotonic Percoll^®^ and centrifuged (1500 rpm, 15 min, 20 °C). The upper cell layer was removed, and pelleted mast cells were washed twice in cDMEM by centrifugation (1200 rpm, 5 min, 20 °C). After washing, mast cells were counted and resuspended in an appropriate volume of cDMEM (for flow cytometric analysis, confocal microscopy, and migration assay) or in medium for rat mast cells, containing 137 mM NaCl, 2.7 mM KCl, 1 mM CaCl_2_, 1 mM MgCl_2_, 10 mM HEPES buffer, 5.6 mM glucose, and 1 mg/ml BSA (for histamine release assay, intracellular Ca^2+^ measurement, and cysLTs and CCL3 synthesis measurements) to obtain a mast cell concentration of 1.5 × 10^6^ cells/ml. Mast cells were prepared with purity > 98%, as determined by metachromatic staining with toluidine blue. The viability of mast cells was over 98%, as estimated by trypan blue exclusion method.

### Histamine release assay

Purified mast cells were resuspended in an appropriate volume of medium for rat mast cells and incubated with leptin at final concentrations of 0.1, 10, 50, or 100 ng/ml, a well-known potent mast cell degranulating factor, i.e., compound 48/80 at final concentration of 5 µg/ml (positive control) or buffer alone (spontaneous histamine release) in a water bath for 30 min at 37 °C with constant stirring. For the time-course experiments, the mast cells were incubated with leptin at a final concentration of 50 ng/ml for 0, 1, 3, 5, 10, or 30 min. After incubation, the reaction was stopped by adding 1.9 ml of cold medium and the release of histamine was measured spectrofluorometrically, as described previously [22].

### Measurement of intracellular calcium

Mast cell suspension (1.25 × 10^5^ cells/ml) was plated into black, 96-well plate. An equal volume of Fluo-4 Direct calcium reagent loading solution was added to each well and plate was incubated at 37 °C for 30 min. Prior to stimulation, baseline fluorescent readings (resting mast cells) were measured from triplicate wells in 1 s intervals for 2.5 min. Mast cells then treated with 50 ng/ml leptin and fluorescent readings were measured in the same mode. The fluorescence monitored in a kinetic mode on Victor ×3 plate reader (Perkin-Elmer) was recorded using 488 nm excitation filter and 535 emission filter. The signal was calibrated by the addition of 0.1% Triton X-100 to obtain maximal signal (*F*_max_) and 10 mM EGTA to record minimal signal (*F*_min_). Changes in Fluo-4 fluorescence were converted to absolute [Ca^2+^]_c_ = *K*_d_ ((*F − F*_min_)/(*F*_max_ *− F*)), where *K*_d_ = 345 nM.

### CysLT synthesis measurement

Purified mast cells were incubated with leptin at final concentrations of 0.1, 10, 50, or 100 ng/ml, with calcium ionophore A23187 at a final concentration of 5 µg/ml (positive control) or in buffer alone (spontaneous cysLT release) in a water bath for 1 h at 37 °C with constant stirring. The supernatants were collected by centrifugation (1200 rpm, 5 min, 20 °C). The concentration of cysLTs in supernatants was evaluated by a commercial ELISA kit, according to the manufacturer’s instructions. The sensitivity of the assay was < 13 pg/ml.

### CCL3 generation measurement

Purified mast cells were incubated with leptin at final concentrations of 0.1, 10, 50, or 100 ng/ml, mouse anti-rat IgE at final concentration of 5 µg/ml (positive control) or buffer alone (spontaneous chemokine generation) in a humidified atmosphere with 5% CO_2_ for 2 h at 37 °C. The supernatants were collected by centrifugation (1200 rpm, 5 min, 20 °C). The CCL3 concentrations in supernatants were evaluated by ELISA, according to the manufacturer’s instructions. The sensitivity of this test was < 59 pg/ml.

### Flow cytometric analysis and confocal microscopy

Constitutive and leptin-induced cell surface CYSLTR1 and CYSLTR2 expressions were determined using flow cytometry and confocal microscopy. Constitutive expression of cysLT receptors was assessed in freshly isolated native mast cells (i.e., non-stimulated cells). Leptin-induced receptor expression was estimated on mast cells incubated with leptin, at final concentrations of 1 or 100 ng/ml, for 1 h in a humidified atmosphere with 5% CO_2_ at 37 °C. In confocal microscopy technique, mast cells were incubated with Tris–HCl under the same conditions (vehicle-treated control). After incubation, the mast cells were fixed with BD CellFIX™ solution for 15 min and washed twice with 1× PBS. Next, the mast cells were resuspended in 1× PBS and stained for 1 h with goat polyclonal IgG N-term antibodies against CYSLTR1 or CYSLTR2 (antibody dilution 1:100). To confirm the specificity of the primary antibody, normal goat IgG antibodies (isotype control) were used. Primary antibody was not added to the sample to certify non-specific binding of the secondary antibody. Cells were then washed with 1× PBS and incubated for 1 h with Alexa Fluor^®^ 488-conjugated secondary antibodies in 1× PBS. Following this, the mast cells were washed twice with 1× PBS and resuspended in 1× PBS.

Ten thousand events in each sample were analyzed using a BD FACSCalibur™ flow cytometer with BD CellQuest™ software (BD Biosciences). Leptin-induced mast cell cysLT receptor expression was presented as a percentage of CYSLTR1 or CYSLTR2 MFI (mean fluorescence intensity) measured in native mast cells (referred to as 100%). After each period of incubation, mast cell viability was examined using the trypan blue exclusion test.

In confocal microscopy, the samples were mounted on microscope slides. Images were captured using an LSM 510Meta confocal laser scanning microscope combined with an Axiovert 200M (Zeiss, Oberkochen, Germany) inverted microscope equipped with a Plan-Neofluar objective (40×/0.6). All settings were held constant throughout the experiments. The fluorescence was recorded using the argon laser (488 nm) and a BP filter set (505 nm). The same laser line was used for Nomarski DIC. All signals obtained from confocal microscopy were validated with profile view image analysis and the diagrams presenting intensity values are placed below each microphotograph. The mean fluorescence intensity is expressed in arbitrary units (AU).

### Mast cell migration assay

The mast cell migratory response to leptin was determined using Boyden microchamber assay in a 48-well chemotaxis chamber as previously described [23]. In brief, 30 µl of leptin (final concentrations from 0.001 to 50 ng/ml), TNF (at final concentrations of 0.01, 0.05, or 0.1 pg/ml; positive control), or buffer alone (control spontaneous migration) was placed into the lower compartments of the microchamber, which were covered with a polycarbonate 8-µm-pore-size membrane. Mast cells were loaded in the upper wells of the Boyden chamber, incubated for 1 h, and migrating cells were detected on the membrane after staining with hematoxylin. Mast cell migration was quantified by counting the number of cells that had traversed the membrane and were attached to the bottom surface of the filter. Ten high-power fields (HPF) were counted in each assay (×250). Spontaneous migration served as a control and was referred to as 100%. The results were presented as a percentage of control migration.

In separate experiments, migration assays were conducted using laminin-coated or fibronectin-coated micropore filters. Filters were coated overnight at room temperature with laminin or fibronectin at a concentration of 100 µg/ml. The filters were air dried for at least 60 min before use.

### Checkerboard analysis

Checkerboard analysis of mast cell migration was performed according to Zigmond and Hirsch [24]. Varying concentrations of leptin were added to the upper and lower wells of the Boyden chemotaxis chamber. Chemotaxis assay was conducted as described, with the use of uncoated filters. Chemotaxis occurred when there was a positive gradient of the chemoattractant. Chemokinetic mobility took place when the chemoattractant was present in both the lower and upper wells at the same concentrations (equivalent concentrations) or when the chemoattractant was present in the upper wells of the chamber (reversed gradient).

### Statistical analysis

The results are expressed as mean value ± standard deviation (SD). Statistical comparisons were performed using Student’s *t* test for small groups. Differences were considered statistically significant at *P* < 0.05 and are labeled with an asterisk (*) on each graph.

## Results

### Leptin induces mast cell degranulation and enhances intracellular Ca^2+^

**Fig. 1:**
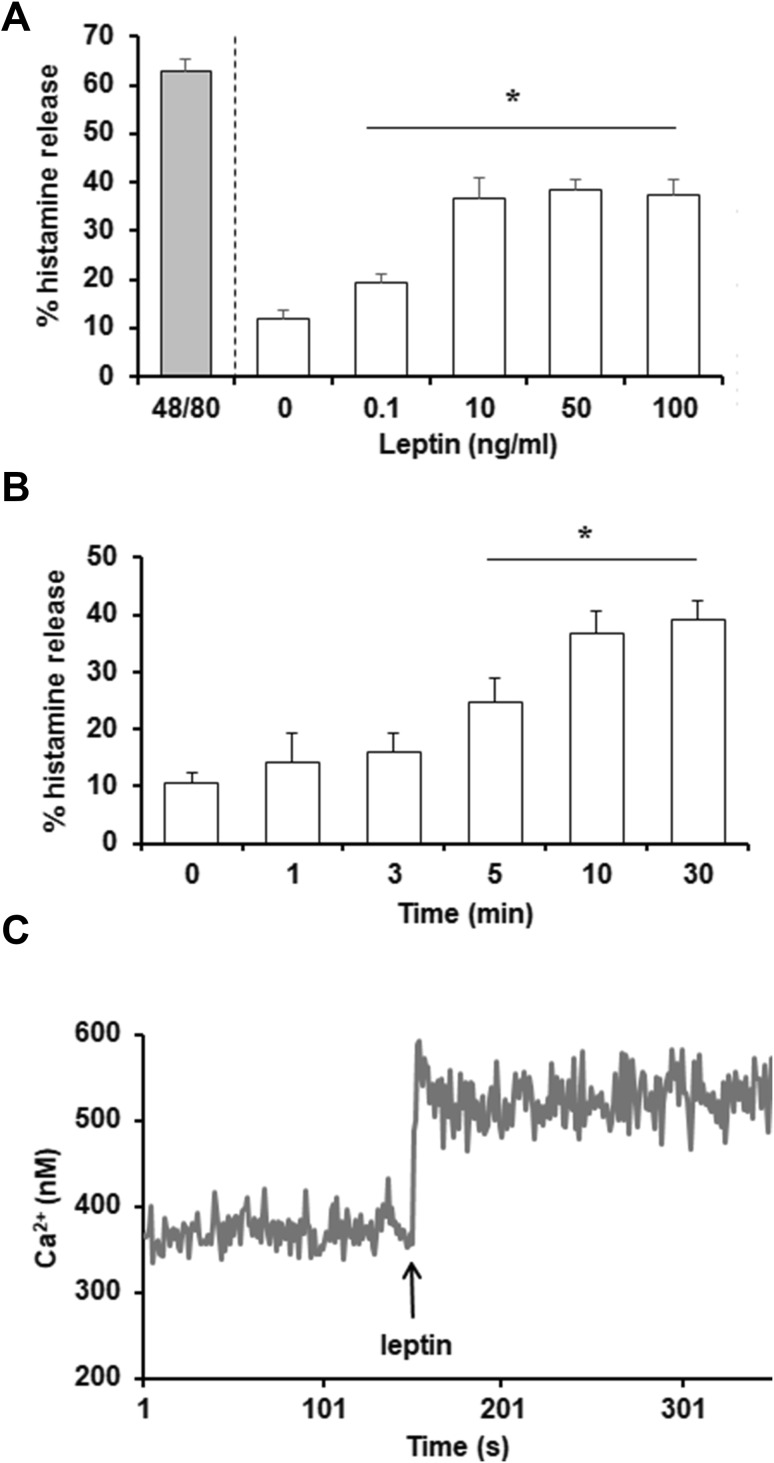
Effect of leptin on mast cell degranulation and intracellular Ca^2+^ level. Mast cells were incubated with different concentrations of leptin, compound 48/80 at 5 µg/ml (positive control), or medium alone for 30 min (**a**). Mast cells were stimulated with leptin at 50 ng/ml at the indicated time periods (**b**). Results are the mean ± SD of three independent experiments and each experiment was done in duplicate. *P < 0.05. The calcium level was determined fluorometrically using the Fluo-4 calcium indicator (**c**). Arrow indicates the addition of leptin at a concentration of 50 ng/ml. Data are the representatives of three independent experiments and each experiment was done in duplicate

The effect of various concentrations of leptin, from 0.1 to 100 ng/ml, on mast cell degranulation and histamine release was evaluated first. Measurements of histamine secretion indicated that this adipocytokine activated mast cell degranulation at all concentrations used (Fig. 1a). Mast cells challenged with leptin at 50 ng/ml released up to 38.3 ± 2.5% of histamine. For comparison, a potent degranulation inducer, i.e., compound 48/80, induced mast cell histamine secretion up to 62.7 ± 2.2% following 30 min of incubation. Time-course experiments revealed that in response to leptin stimulation, slight histamine release was observed as early as 1 min; however, statistically significant secretion occurred within 5 min (Fig. 1b). After 30 min of stimulation, leptin-induced histamine release had increased up to 39.0 ± 3.5%.

We next examined the effect of leptin on the intracellular Ca^2+^ level using Fluo-4-loaded mast cells. We found that leptin, at a concentration of 50 ng/ml, induced an increase of intracellular Ca^2+^ level in mast cells within 10 s after stimulation, compared to resting mast cells (Fig. 1c). After initial rise, intracellular Ca^2+^ level reached a plateau phase.

### Leptin activates mast cells to cysLTs and CCL3 generation

**Fig. 2:**
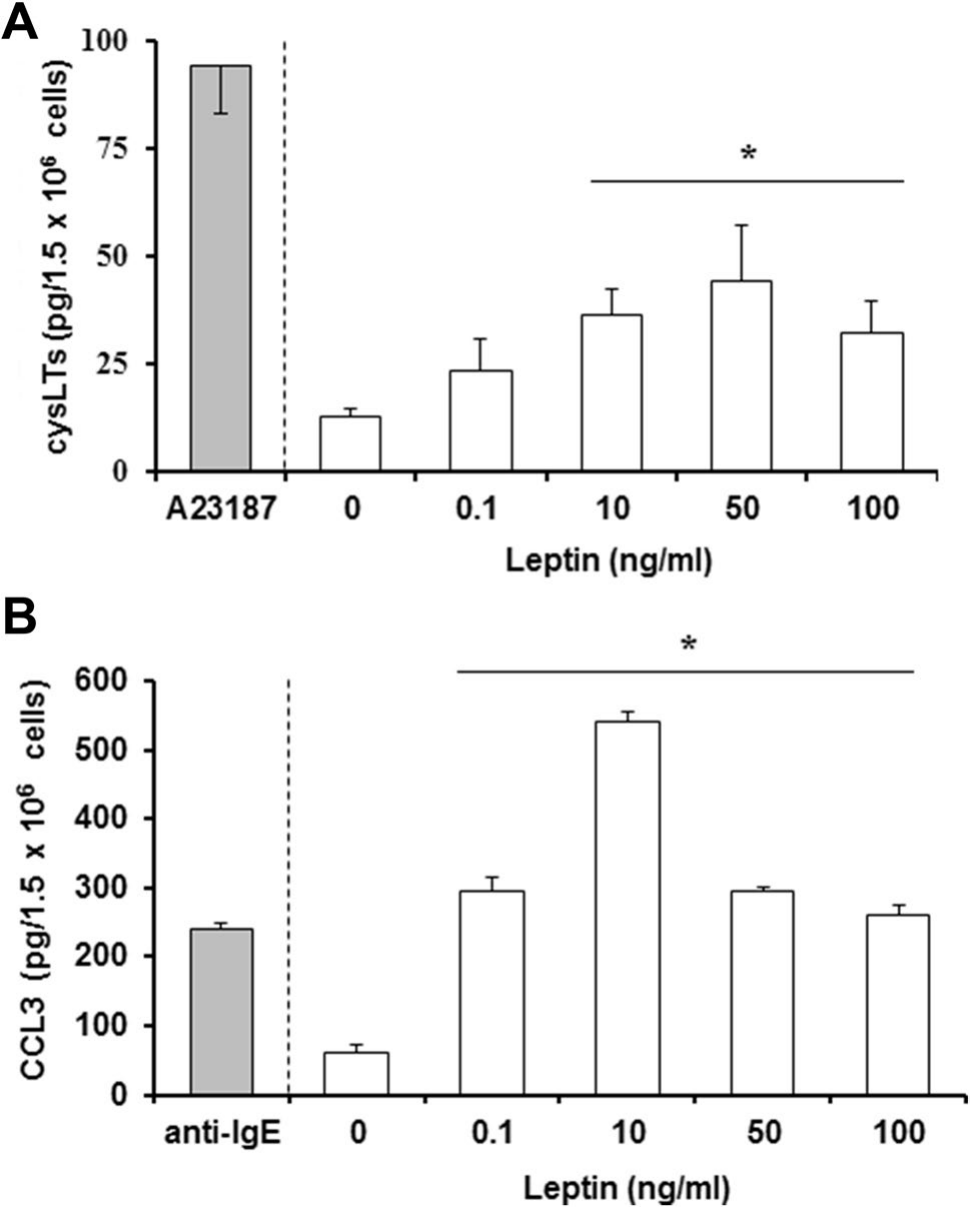
CysLTs and chemokine CCL3 released by mast cells following exposure to leptin. Mast cells were incubated with leptin at different concentrations, calcium ionophore A23187 at 5 µg/ml or anti-IgE at 5 µg/ml (positive controls), or medium alone and levels of cysLTs (**a**) or CCL3 (**b**) were measured in supernatants by ELISA. Results are the mean ± SD of four independent experiments done in duplicate. *P < 0.05

The next stage investigated whether leptin can directly activate mast cells to generate and release newly synthesized arachidonic acid metabolites, i.e., cysLTs. As shown in Fig. 2a, leptin stimulation caused dose-dependent cysLTs generation by mast cells, and at leptin concentration of 50 ng/ml, rat mast cells released up to 44.3 ± 15.9 pg cysLTs/1.5 × 10^6^ mast cells. In comparison, cysLTs generation and release after ionophore A23187-stimulation was as high as 94.3 ± 15.5 pg cysLTs/1.5 × 10^6^ mast cells. In addition, significantly greater amounts of chemokine CCL3 were synthesized and released from mast cells stimulated with leptin than those stimulated with anti-IgE (Fig. 2b). The mast cells released up to 540 ± 14.0 pg CCL3/1.5 × 10^6^ mast cells following exposure to 10 ng/ml leptin, compared to 240 ± 10.0 pg CCL3/1.5 × 10^6^ mast cells following anti-IgE stimulation.

### Leptin affects surface CYSLTR1 and CYSLTR2 protein expression on mast cells

**Fig. 3:**
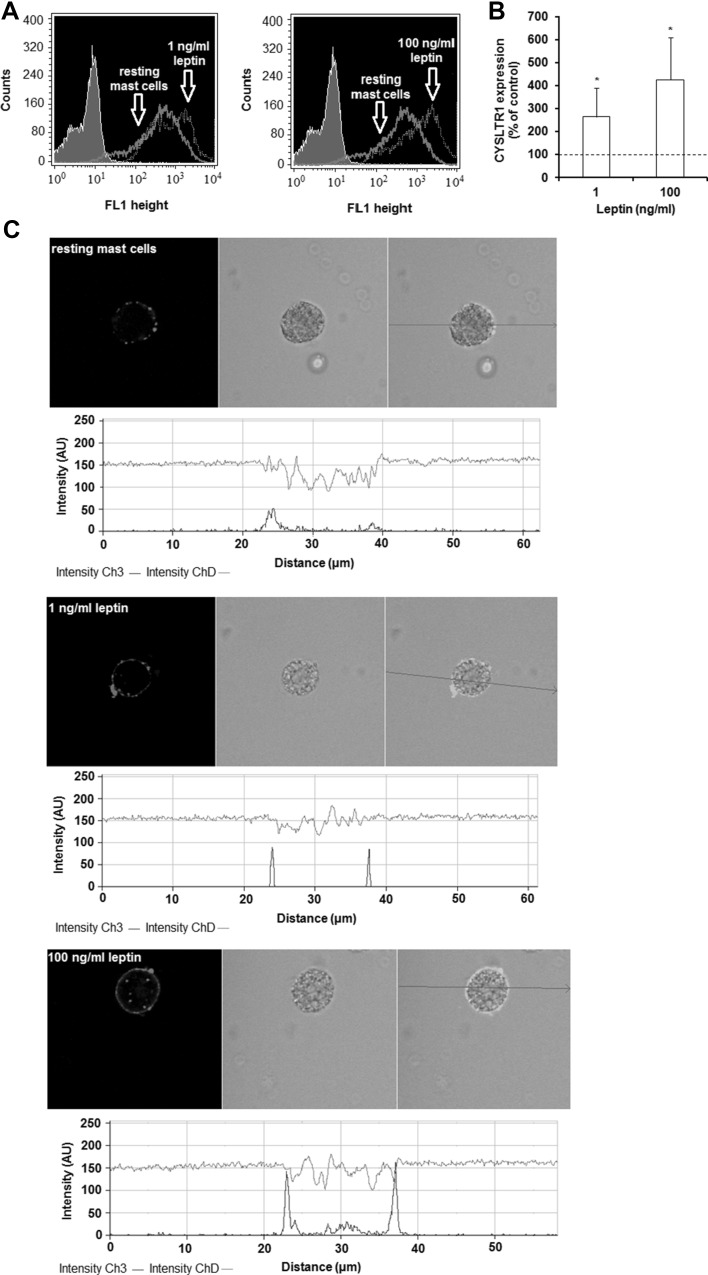
Effects of leptin stimulation on CYSLTR1 expression in mast cells. Mast cells were incubated with medium alone, leptin at 1 or 100 ng/ml for 60 min. The signal was visualized with green Alexa488. Constitutive and leptin-induced at 1 or 100 ng/ml surface CYSLTR1 protein expression assessed by flow cytometry. Shaded tracings - isotype control, open tracings—CYSLTR1 expression in resting (green) and leptin-induced (red) cells (**a**). Constitutive CYSLTR1 protein expression served as a control and was referred to as 100%. The results are presented as percentage of constitutive CYSLTR1 protein expression. Results are the mean of fluorescent intensity ± SD of three experiments performed in duplicate. *P < 0.05 (**b**). Representative images showing surface CYSLTR1 expression analyzed by confocal microscopy in resting cells, leptin-induced cells at 1 ng/ml, and leptin-induced cells at 100 ng/ml (**c**). Below each microphotograph fluorescence intensity diagrams, in arbitrary units (AU), showing the distribution of fluorescence in cells was mounted. (Color figure online)

The next stage examined whether leptin stimulation influences CYSLTR1 and CYSLTR2 expression by mature rat mast cells. The constitutive and leptin-induced surface expression of CYSLTR1 in mast cells, as measured using flow cytometry, is shown in Fig. 3a. The baseline level of CYSLTR1 expression was found to be significantly up-regulated (*P* < 0.05) upon incubation with 1 or 100 ng/ml leptin, reaching 263.6 ± 127.2 and 425.6 ± 182.9% of control CYSLTR1 expression on native mast cells, respectively (Fig. 3b). These findings are in good agreement with the confocal microscopy analysis (Fig. 3c). The fluorescence intensity diagrams beside each microphotograph confirm that mast cell stimulation with leptin at concentrations of 1 or 100 ng/ml resulted in, respectively, 188% or 288% greater CYSLTR1 surface expression compared with native cells.

**Fig. 4:**
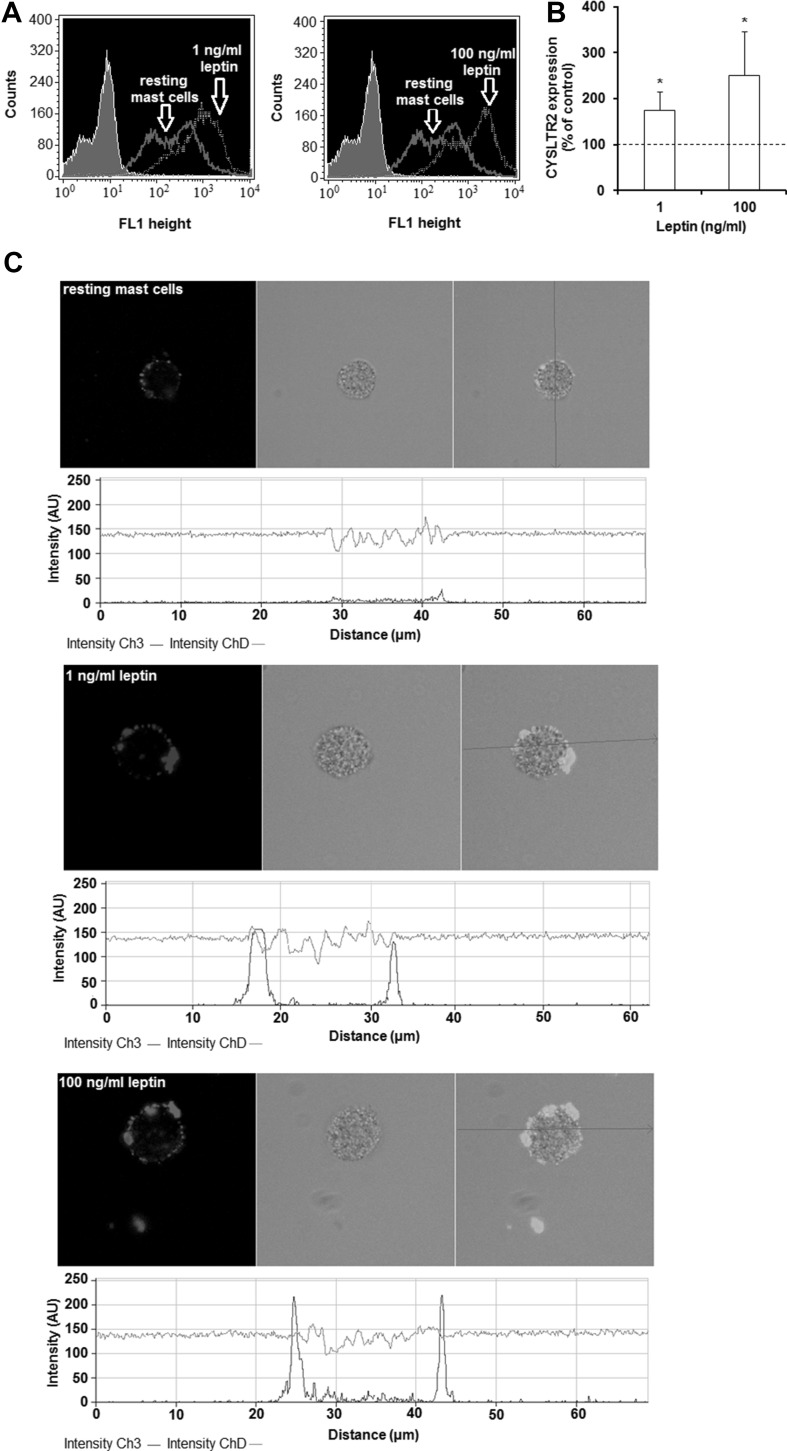
Effects of leptin stimulation on CYSLTR2 expression in mast cells. Mast cells were incubated with medium alone, leptin at 1 or 100 ng/ml for 60 min. The signal was visualized with green Alexa488. Constitutive and leptin-induced at 1 or 100 ng/ml surface CYSLTR2 protein expression assessed by flow cytometry. Shaded tracings—isotype control, open tracings—CYSLTR2 expression in resting (green) and leptin-induced (red) cells (**a**). Constitutive CYSLTR2 protein expression served as a control and was referred to as 100%. The results are presented as percentage of constitutive CYSLTR2 protein expression. Results are the mean of fluorescent intensity ± SD of three experiments performed in duplicate. *P < 0.05 (**b**). Representative images showing surface CYSLTR2 expression analyzed by confocal microscopy in resting cells, leptin-induced cells at 1 ng/ml, and leptin-induced cells at 100 ng/ml (**c**). Below each microphotograph fluorescence intensity diagrams, in arbitrary units (AU), showing the distribution of fluorescence in cells was mounted. (Color figure online)

The changes in CYSLTR2 expression level are shown in Fig. 4a. Mast cell stimulation with leptin used at a concentration of 1 ng/ml resulted in a statistically significant (*P* < 0.05) increase in CYSLTR2 level, compared with the control unstimulated mast cells (174.4 ± 40.2% of control CYSLTR2 expression). Likewise, a higher leptin concentration (100 ng/ml) induced an enhancement of CYSLTR2 protein level, reaching 250.9 ± 95.4% of control CYSLTR2 expression on native mast cells (Fig. 4b). To confirm these results, single confocal sections with fluorescence intensity diagrams were analyzed (Fig. 4c). It was found that mast cell stimulation with 1 ng/ml of leptin resulted in an eightfold increase in CYSLTR2 expression compared with non-stimulated cells. Image analysis also demonstrated that the baseline level of cell surface CYSLTR2 was 12.5-fold up-regulated upon incubation with leptin at a concentration of 100 ng/ml. In both techniques, the specificity of the antibodies was confirmed by isotype controls and controls for non-specific binding of the secondary antibody (data not shown). The vehicle control showed that solvent used for leptin had no impact on the obtained results.

### Leptin stimulates mast cell migratory response

**Fig. 5:**
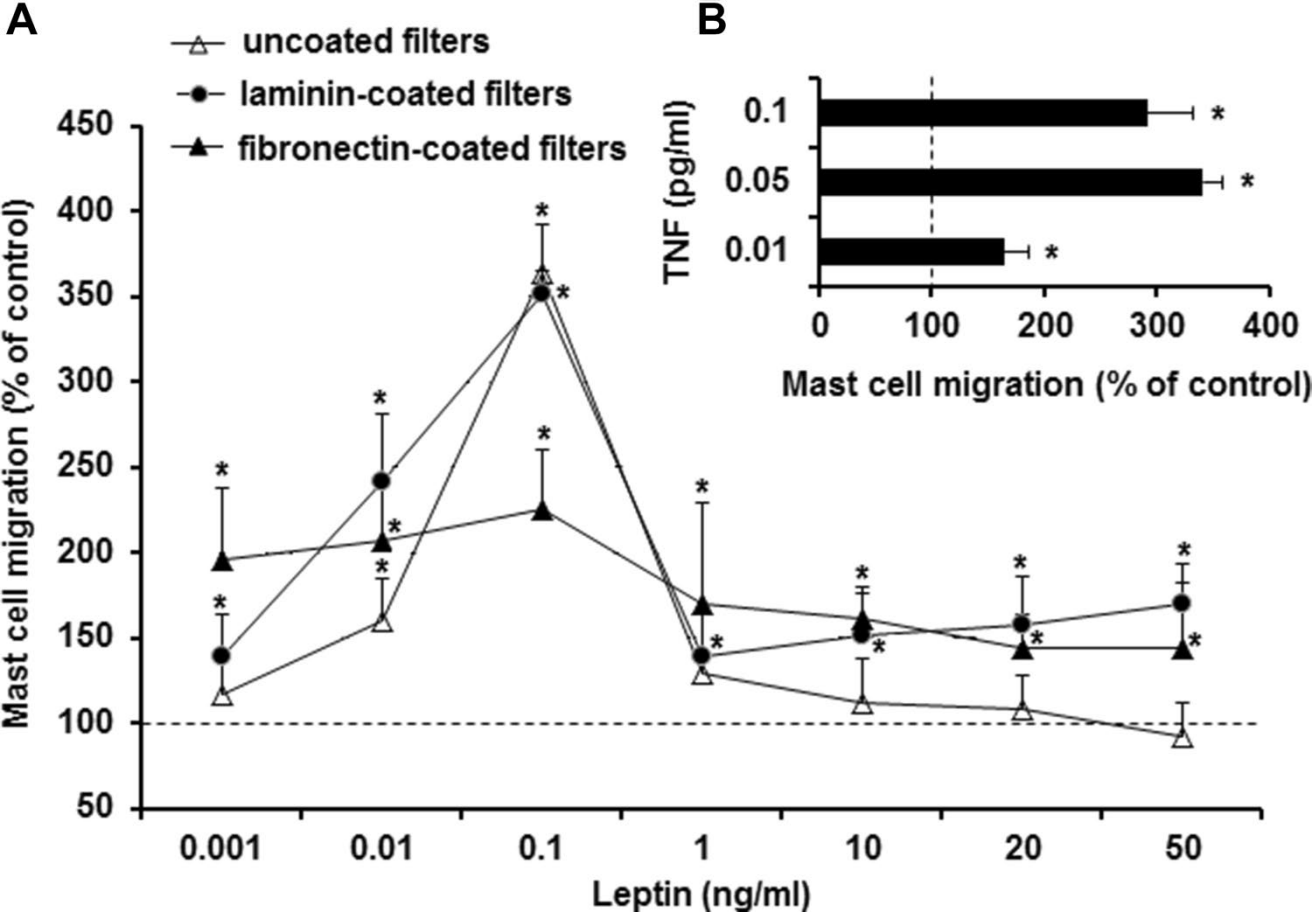
Leptin-induced (**a**) and TNF-induced (**b**) mast cell migration. Mast cells were incubated with different concentrations of leptin, TNF (positive control) or medium alone (control spontaneous mast cell migration) for 3 h at 37 °C in a Boyden microchamber. Ten HPF were counted in each assay (×250). Spontaneous migration served as a control and was referred to as 100%. Each point represents the mean ± SD of three independent experiments done in duplicate. *P < 0.05

The study also examined the ability of various concentrations of leptin to induce migration of rat tissue mast cells. The results were compared with those obtained for the migration of mast cells in response to TNF, a well-known and robust, mature mast cell chemotactic factor [25]. Leptin was found to induce dose-dependent migration of mast cells, even in the absence of the ECM proteins, laminin, and fibronectin. The optimal leptin concentration for maximal mast cell migration was 0.1 ng/ml (Fig. 5a). Interestingly, higher concentrations of leptin, ranging from 1 to 50 ng/ml, induced nonsignificant mast cell migration. In the presence of laminin or fibronectin, only slightly enhanced mast cell migration was observed, especially when leptin was used at higher concentrations. For comparison, rat mast cells also migrated in response to TNF stimulation under the same experimental conditions, with the optimal concentration of TNF for maximal migration of mast cells being 0.05 pg/ml (Fig. 5b).

The migration of mast cells was then examined in the presence of positive and negative leptin gradients to determine whether this adipocytokine promotes chemotaxis (directional migration) or chemokinesis (random migration). Checkerboard analysis with varying leptin concentrations in both the upper and lower wells of the chemotaxis microchamber showed that leptin-induced gradient-dependent migration of mast cells, i.e., chemotaxis (Table 1).

**Table 1:** Checkerboard analysis of leptin-induced mast cell migratory response

Leptin in lower compartment (ng/ml)	Leptin in upper compartment (ng/ml)			
	0	0.01	0.1	1
0	100 ± 25	99 ± 10	104 ± 11	86 ± 5
0.01	146 ± 9*	95 ± 10	94 ± 4	91 ± 6
0.1	318 ± 22*	108 ± 6	113 ± 6	91 ± 7
1	127 ± 9	109 ± 8	101 ± 8	90 ± 8

Mast cells were incubated with different concentrations of leptin or medium alone (control spontaneous mast cell migration) for 3 h at 37 °C in a Boyden microchamber. Ten HPF were counted in each assay (×250). Spontaneous migration served as a control and was referred to as 100%

Each point represents the mean ± SD of three independent experiments done in duplicate (*n* = 6)

*P < 0.05

## Discussion

There is some information that mast cells express leptin receptor. This receptor has been demonstrated on immature murine bone marrow-derived mast cells (BMMCs) [26]. Taildeman et al. [15] documented the presence of this receptor in human tissue mast cells. Immunofluorescence staining showed mainly cytoplasmic, granular localization of leptin receptor in these cells; however, it was also expressed in the cytoplasmic rim lining the cell surface membrane. It has, therefore, been suggested that leptin might exert an immunomodulatory effect on mast cells. Nevertheless, the current understanding of the direct action of leptin on mast cells remains unclear. Only Zhou et al. [26] documented that leptin induces histamine release as well as IL-13, IFN-γ, and CCL2 production, and reduces IL-4, IL-6, IL-10, and CCL3 synthesis by murine BMMCs. Hence, the present study aimed to investigate the effect of leptin on matured in vivo rat mast cells isolated from the peritoneal cavity, that is, connective tissue-type mast cells.

We documented that mast cell activation with leptin led to dose-dependent and time-dependent mast cell degranulation and histamine release. Simultaneously, leptin induced the intracellular Ca^2+^ increase, as well. We also established that leptin stimulation resulted in cysLTs and CCL3 generation by mast cells. Histamine and cysLTs are known to be one of the most potent mediators that play a crucial role in inducing and maintaining inflammatory processes. Especially, these mediators stimulate different cells to the production of pro-inflammatory mediators and cytokines, promote cell adhesion to the vascular epithelium, and have the potency to recruit immune cells to the site of inflammation. Histamine and cysLTs increase vascular permeability, as well. Chemokine CCL3 promotes tissue fibrosis and engages in activation and recruitment of neutrophils, macrophages, and lymphocytes to the site of inflammation. Moreover, we found that leptin causes enhancement of CYSLTR1 and CYSLTR2 surface expression on mast cells. These results are intriguing, as they might suggest that leptin enhances mast cell sensitivity to cysLTs.

An important finding is that leptin can act as a chemoattractant for fully mature tissue mast cells. It is well documented that while mast cell numbers are high in tissues, they remain relatively constant under physiological conditions. In contrast, mast cell number can increase dramatically in local tissues during various pathological conditions, including acute and chronic inflammation [4, 10]. It is also important to note that mast cells occur in great numbers within adipose tissue, adjacent to leptin-produced adipocytes, and their number within this tissue is raised in obese individuals [27]. Moreover, there is a suggestion that mast cells are involved in low-grade inflammation within adipose tissue [28].

It should be underlined that so far the physiological levels of leptin have not been precisely determined. In our in vitro studies, we used leptin at concentrations in the range 0.1–100 ng/ml. There are data that blood plasma concentrations of leptin are 11.6 ng/ml in healthy subjects, and 34.7 ng/ml in patients with obesity [29]. Bozan et al. [30] found serum levels of leptin to be 7.82 ng/ml in non-obese, and 72.1 ng/ml in obese children. Therefore, we assume that leptin concentrations used in our study reflect the leptin concentrations present in pathophysiological conditions.

A growing body of data supports that activity of many immune/inflammatory cells is modulated by leptin, a well-known member of the adipocytokines. Macrophages exposed to leptin produce TNF, IL-6, IL-1β, IL-1ra, CCL2, and CCL3, as observed for M1 cells [31]. Leptin pretreatment of macrophages also increases the synthesis of lipid mediators, i.e., cysLTs, LTB_4_, and PGE_2_ in response to calcium ionophore and zymosan [32]. This adipocytokine induces the release of the pro-inflammatory cytokines IL-1β and IL-6, and the chemokines CCL2, CXCL1, and CXCL8 by eosinophils [33]. It directly induces basophil degranulation and histamine release and stimulates IL-4 and IL-13 synthesis in these cells [34]. Leptin acts as a survival factor for neutrophils [35] and eosinophils [36], as it delays their inevitable death. In addition, leptin was observed to have a significant impact on neutrophil and monocyte reactive oxygen species (ROS) generation [37, 38]. Leptin increases both the development and the activation of NK cells [39] and inhibits the production of IL-10 by these cells [40]. Importantly, leptin promotes the migration of basophils, monocytes/macrophages and endothelial cells [34, 41, 42], as well as of eosinophils and neutrophils [33, 43]. Our observations clearly documented that leptin promotes the pro-inflammatory activity of mast cells, and thereby it engages these cells in the inflammatory processes.

It is important to note that mast cells contain leptin within cytoplasmic granules, and it has been suggested that leptin release co-occurs with the release of neutral proteases, i.e., tryptase or chymase, and may be controlled by the same factors that regulate mast cell activation and degranulation [15]. Therefore, one can assume that leptin could act both as a paracrine and autocrine regulator of mast cell activity. Indeed, further studies are needed to clarify the influence of leptin on mast cell biology within tissues.
